# Different strategies, but indifferent strategy adaptation during action cascading

**DOI:** 10.1038/srep09992

**Published:** 2015-05-07

**Authors:** Moritz Mückschel, Ann-Kathrin Stock, Christian Beste

**Affiliations:** 1 Cognitive Neurophysiology, Department of Child and Adolescent Psychiatry, Faculty of Medicine of the TU Dresden, Germany

## Abstract

Every day, we need to apply different action control strategies to successfully interact with ever-changing environments. In situations requiring several responses, we often have to cascade different actions. The strategies used to accomplish this have been subject to extensive research in cognitive psychology and neuroscience but it has remained rather unclear if and to what degree such strategies are adapted while performing a task. Furthermore, we do not know if such adaptations are subject to differential effects depending on an individual’s preferred initial strategy to cope with multiple-demand situations. Using Bayesian analyses, we were able to show that even though the applied strategy is subject to slight modulations over the course of an action cascading task, this shift is equally strong for subjects who differ their general action cascading strategy. The action cascading strategy subjects apply to cope with multiple-demand situations is adapted independent of the preferred, inter-individually varying strategy that is initially used. Future research needs to test if the task goal activation strategy applied during action cascading reflects a ‘cognitive trait’ and is stable across different situations.

In everyday life, we are frequently faced with complex situations where response control is required in a multitude of different tasks so that we have to chain or cascade different actions in order to successfully interact with our environment. Over the last decades, the strategy we use to accomplish this has been subject to extensive research. It could be shown that the modes used to cascade different actions and perform response selection in action cascading/dual-tasking situations range from a more serial to a more parallel processing strategy^1^,^2^. In a more serial strategy, a task goal is usually not activated until the previous one has been carried out, while in a more parallel strategy, a task goal may become activated while the previous one is still being carried out^2^. In this context, it however needs to be pointed out that we usually do not display a “purely” serial or parallel strategy. Instead, we tend to allocate our strategy somewhere in between those two poles, resulting in a varying degree of overlap between different task goal processes^1^,^2^.

However, parameters calculated to classify the response selection strategy applied in dual-tasking and action cascading are usually based on overall performance in the entire experiment^1^,^2^,^3^,^4^,^5^,^6^,^7^. Using such measures hence implies that the derived parameter is representative for the entire experiment. Yet, it is well-known that training may affect the response selection mode applied in dual-tasking situations^8^. It is therefore likely that the strategy used to activate task goals changes with time or may be adapted over the course of the experiment. Until today, it has however remained elusive whether such changes are comparable for everybody or whether individuals with different task goal processing strategies also display differently large shifts in their respective strategies^4^. We therefore investigated individual strategy shifts over time to find out whether the applied strategy is robust against modulatory effects of adaptation mechanisms occurring over the course of a task.

For this purpose, we employed a stop-change task introduced by Verbruggen *et al.*^2^ which employs two stimuli triggering two different actions, namely stopping a right-hand response and executing a left-hand response. Its most relevant feature is that those inputs are either presented with a temporal gap of 300 ms (with a so-called stop-change delay/SCD of 300 ms) or presented simultaneously (termed “SCD0”). While serially presenting stimuli enforces serial processing of the associated task goals, a simultaneous stimulus presentation leaves participants with the “choice” of how (rather serially or more in parallel) they process their task goals. This should affect both response times (RTs) in the SCD0 condition as well as a slope parameter plotting the response time difference of the two SCD conditions over the temporal delay with which the stimuli are presented. Eventually, both parameters (SCD0 RTs and the slope parameter) can be used to estimate the degree of task goal overlapping^2^,^9^ (i.e. the applied action cascading strategy, please see methods section for more details).

Our study question raises important methodological considerations: If the applied strategy is adapted independent of the preferred, inter-individually varying strategy chosen to cope with multi-demand situations (i.e. subjects classified into different “strategy groups” do not show a differential modulation of response selection strategy across sections of the experiment), this would necessitate accepting the null hypothesis. It is therefore indispensable to examine the probability of the null hypothesis being true, given the obtained data (*p*(H_0_|D)). Besides classical null hypothesis testing (NHST) using mixed effects ANOVAs, we therefore also used Bayesian statistics^10^,^11^ to evaluate the relative strength of evidence for the null and alternative hypothesis. Using the Bayesian approach, it is possible to provide a quantification of the degree to which the data supports the null hypothesis^10^.

## Results

**Figure 1:**
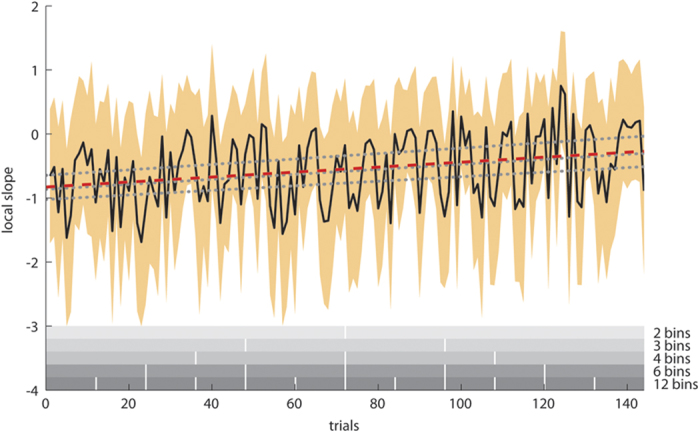
Changes of the single-trials slope parameter (y-axis) of the SCD-RT function plotted against the trial number/order of trials during the experiment (x-axis). The mean shift of the parameter (mean for all subjects) is denoted by the black line. The orange area denotes the standard deviation, the red dashed line the overall trend of shift of the over the course of the experiment. The grey dotted lines denote the mean group trends of shift for an exemplary partitioning into three groups. The grey scale horizontal bars at the bottom denote the different bins used to analyse the shift of the slope parameter.

The single-trial slopes (obtained using the SCD-RT function described in the methods section) are shown in Fig. 1 for the overall cohort as a function of trial number. For data analysis, we formed bins across the 144 experimental SCD0 trials with simultaneous input. We began with 2 bins comparing the first 50% of trials to the last 50% of trials. We then gradually increased the number of bins up to 12 bins. The numbers of bins used to subdivide the experiment were 2, 3, 4, 6 and 12 (all common divisors of the 144 SCD0 trials used in the study). The bins were used to analyse if and how the slope parameter (which was calculated for each trial, see methods section) and hence the applied action cascading strategy changed over the course of the experiment. To investigate whether it is differentially modulated across those subjects using a more serial and those using a more parallel task goal processing strategy, subjects were also classified into 2 to 5 different groups, based on their overall slope (calculated using mean RTs for all SCD0 and SCD300 trials). We used different numbers of groups to examine if differences are evident when considering opposing extremes of the strategy applied during action cascading. This also avoids the possibility that obtained effects are caused by the artificial group classification. Fig. 1 shows the regression lines for the 3-group partition of the entire sample used in the experiment. As can be seen in Fig. 1, the regression lines of all 3 groups were parallel to each other, yet the intersection with the y-axis differed between the groups (F(2,247) = 22.99; p < .001; η^2^ = .157). All groups differed from each other (p < .018). This example shows that the groups we formed differ in the overall strategy they applied. This validates the group partition used to examine the study questions.

There was no difference between groups in terms of reaction times (RTs) on GO trials (all F < 1.2p > .6). We further calculated mixed effects ANOVAs for each combination of bin number and number of groups. All ANOVAs showed that the slope parameter and hence the applied strategy was instable and thus changed over the course of the experiment (all F > 83.01; p < .001; η^2^ > .251; see Fig. 1). Furthermore, there was always a main effect of group (all F > 13.48; p < .001; η^2^ > .180), with all groups differing from each other, even when 5 groups were used (p < .001). This shows that the “strategy groups” indeed show distinguishable modes of action cascading. However, in none of the performed mixed effects ANOVAs, there was an interaction of “bin x group” (all F < 0.38; p > .5). For all conducted ANOVAs, the observed power was β > .98. The non-significant interaction of “bin x group” suggest that there were no differences in the modulation of the slope parameter between subjects classified as belonging to different groups. Groups showing differences in the strategy of task goal activation during action cascading (as reflected by the overall slope) therefore seem to show similar fluctuations in their respective cascading strategies. This does however not exclude, that the individuals in the groups showed larger fluctuations going in opposite directions. We therefore correlated the slopes in the different bins with each other. All correlation analyses showed that there were substantial correlations between the slope values in two consecutive bins (all p < .01; r > .42). However, no correlations were obtained when bins other the consecutive ones were used in the correlation analyses (all p > .4; r < .10). This suggests that the slope (strategy) shows stability for shorter time periods of the experiment, but not across the entire experiment.

**Figure 2:**
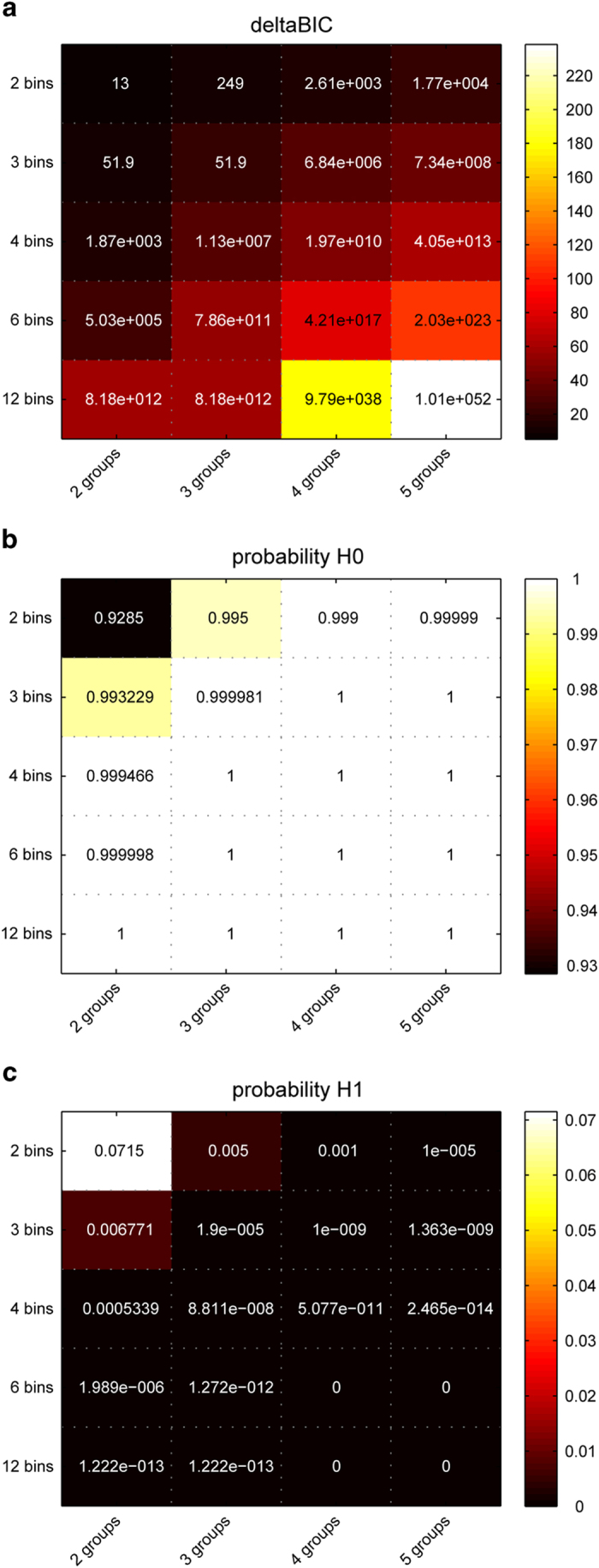
Plots depicting the results of the Bayesian analyses. Each plot denotes a parameter of the Bayesian analysis as a function of the number of bins into which we divided the experiment and the number of classification groups. The colour coding in figure part (**A**) shows the delta-BIC parameter, the values inserted in the heat map the BF parameter. The heat maps in figure parts (**B**) and (**C**) denote the probability of the H_0_ (p_BIC_(H_0_|D)), or H_1_ (p_BIC_(H_1_|D)) being true, respectively. As can be seen, the probabilities in plots (**B**) and (**C**) add up to 1. Warm colours denote higher values or probabilities for the different parameters.

We used Bayesian statistics to analyse the finding that there were similar fluctuations in all groups even though they applied different action cascading strategies in more depth and to evaluate the strength of evidence for the finding that there seemed to be no group-dependent modulation. On the basis of the sum of squares of the error term and the effect term provided by the ANOVAs, we estimated the Bayes factor (BF) for each interaction effect using the Bayesian information criterion (BIC) as proposed by Wagenmakers^11^. The BF can then be converted into the posterior probability that the data favour the null hypothesis (*p*_BIC_(H_0_|D)) by calculating BF/(BF + 1)^10^. The posterior probability that the data favour the alternative hypothesis (*p*_BIC_(H_1_|D)) is calculated as 1-*p*_BIC_(H_0_|D). The results of the Bayesian analyses are shown in Fig. 2.

Regardless of the number of bins used to analyse fluctuations in the strategy of task goal activation and regardless of the partitioning of subjects into different groups, the Bayesian statistics show that according to Raftery^12^, there is strong and very strong evidence in favour of the null hypothesis.

## Discussion

The results provide evidence that the strategy used during action cascading is modulated across the experimental session. As can also be seen in Fig. 1 (i.e. the slope parameter), there is a stronger overlap between task goals activated during action cascading at the beginning of the experiment (i.e. a more parallel cascading strategy is applied in the overall sample). Over the course of the experiment, this overlap becomes weaker (i.e. the applied processing mode becomes more serial in the overall sample). This observation is well in line with findings on training effects during dual-tasking^8^ which show that the response selection strategy is adapted during task performance. However, this modulation seems to be independent of the nature of the subjects’ (initial) preferred processing strategies because the degree of change in the individual processing strategies did not differ across groups. Given that the results did not change when differently composed subject groups or time bins were used, the overall strategy could be regarded as a stable strategy for each individual even though it is clearly subject to dynamic changes. In other words, individuals who initially chose a more serial strategy still chose a more serial strategy at the end of the task, as compared to individuals who initially chose a more parallel strategy. This overall strategy was then gradually adapted, but this happened with a similar magnitude for each individual and independent of the overall initial or terminal strategy. The correlation analyses underline this interpretation, i.e., they suggest that the slope (strategy) shows stability for shorter time periods of the experiment, but not across the entire experiment. Bayesian analyses show that there is strong evidence to assume that the individual shift in applied action cascading strategies is similar in different groups. From the perspective of inter-individual differences, the results suggest that the mode which subjects apply to cope with multiple-demand situations and perform action cascading is similarly adapted in all investigated individuals (i.e. independent of the preferred, inter-individually varying strategy to cope with multi-demand situations). This adaption process may operate in a similar manner/mode for all individuals, reflecting a universal cognitive mechanism, independent of interindividual strategy differences.

This is well in line with studies examining the neural basis of these action cascading strategies. In such studies, the overall slope value describing and quantifying the action selection strategy has been used^3^,^4^,^5^,^6^. These studies have been shown to reveal robust effects regarding several neurobiological modulators. Such findings were unlikely to occur if the overall slope parameter was merely modulated by only a fraction of the task’s trials (e.g. at the end of the experiment). – If this parameter just reflected noisy data, it would be very unlikely to show substantial effects when tested for the modulatory effects of different neurobiological and functional neuroanatomical factors.

In summary, our study shows that the task goal activation strategy used during action cascading is adapted during task performance. However, this adaptation is similar across all subjects, regardless of the different (initial or overall) action cascading strategy applied to cope with multi-demand situations. Future research needs to test if the overall task goal activation strategy applied during action cascading may be stable for each individual across different situations and therefor reflect a ‘cognitive trait’. Currently, we are only on the verge of understanding the neurobiological basis of this trait^3^,^5^,^6^,^13^,^14^,^15^. Future research should address how this trait relates to other factors describing stable inter-individual differences.

## Methods

### Ethics statement

The study was carried out in accordance with the World Medical Association Declaration of Helsinki and the guidelines for experimentation with humans by the Faculty of Medicine at TU Dresden. The experimental protocol was approved by the Ethics Committee of the Faculty of Medicine at TU Dresden (Germany) and the Ethics Committee of the Ruhr University Bochum (Germany). All participants gave written informed consent before the study began.

### Participants

For this study, we gathered data from n = 250 subjects (131 females). The mean age of the subjects was 23.83 and ranged between 18 and 30 years. All participants had normal or corrected-to-normal vision and no hearing impairments. Participants either received course credits or financial compensation for their participation. Based on their task performance, subjects were classified as processing task goals either more serially or more in parallel (as already described in Mückschel *et al.*^4^; please see “calculation” text section for further details).

### Task

**Figure 3:**
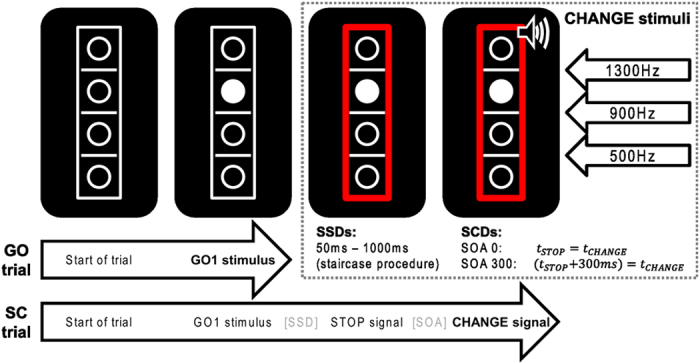
Schematic illustration of the applied SCD paradigm (cf. Stock *et al.*, 2014b) as described in the text.

The task is shown in Fig. 3.

Participants were seated in a dimly lit and sound-attenuated room. Stimuli were presented on a 17” CRT monitor and via headphones with the help of Presentation (v. 14.9, Neurobehavioral Systems, Inc.). Responses had to be given with the help of four buttons located on two custom-made response devices.

The task was a modified version of the stop-change paradigm by Verbruggen *et al.*^2^. Each participant completed an extensive training block prior to the experiment. At the start of each trial, a rectangular frame (20 × 96 mm) containing four vertically aligned circular frames (8 mm diameter) and three horizontal reference lines (line thickness: 1 mm; width: 8 mm) separating the circles were presented on black background in the centre of the screen. After 250 ms, one of the circles was filled with white colour, thus turning into the GO stimulus (target). In the Go trials (2/3 or 67% of trials), participants were instructed to indicate whether the target was located above or below the middle reference line. Responses were given by pressing the outer right key with the right middle finger (“above” judgment) or by pressing the inner right key with the right index finger (“below” judgment) on one of the two the response devices. The stimuli remained on the screen until the participant responded. In case of responses longer than 1000 ms, the German word “Schneller!” (engl. “Faster!”) was presented above the box until the participant responded and thereby ended the trial. 1/3 or 33% of the trials were stop-change (SC) trials. Like GO trials, SC trials started with the presentation of the empty array followed by the target. After a variable stop signal delay (SSD), a STOP signal (a red rectangle replacing the white frame) was presented. Upon its appearance, participants were required to try to inhibit their right hand response to the GO stimulus. The SSD was initially set to 450 ms and adapted to the participants’ performance by means of a ‘staircase procedure’^2^ yielding a 50% probability of successfully inhibited GO responses. The STOP signal was followed by a CHANGE stimulus, which was a 100 ms sine tone (75 db SPL) presented via headphones. It could be either high (1300 Hz), medium (900 Hz) or low (500 Hz). The stop-change delay (SCD) between the STOP and CHANGE signals was either 0 ms (SCD 0) or 300 ms (SCD 300). Each of the tones coded for one of the reference lines (high tone=high line medium tone=medium line, low tone=low line) so that the CHANGE signal in SC trials “assigned” a new reference line. All three tones/reference lines were in effect equally often. The required CHANGE response had to be performed with the left hand. If the target was above the newly indicated reference line, an outer left key press (left middle finger) was required and if the target was located below the newly assigned reference line, a left inner key press (left index finger) was required. In case of response times (RTs) longer than 2000 ms, the German word “Schneller!” (translates to “Faster!”) was presented above the box until the participant responded to end the trial. After each SC trial, the SSD for the next SC trial was adjusted by the above-mentioned staircase algorithm^16^: In case of a correct response (defined as a correct inhibition of GO response *and* a correct CHANGE response), the SSD was prolonged by 50 ms, otherwise shortened by 50 ms. Due to this adaptive procedure, subjects respond incorrectly or prematurely in roughly half of the SC trials. To keep the experiment within reasonable limits, SSDs were confined to the range of 50 - 1000 ms. Each trial was followed by an inter-trial interval of 900 ms. Participants were instructed to respond as fast and accurately as possible. The experiment consisted of 864 trials divided into 6 blocks. The trial order was pseudo-randomized.

### Calculation

The SCD of either 0 or 300 ms is critical for the estimation of the task goal processing strategy^2^,^9^ because the stochastic variation of trial types (GO, and SC with SCD0 or SCD300) results in a mixture of trials in which the process of inhibiting the GO response is sometimes terminated before the CHANGE signal is presented (SCD300 condition) and processed while at other times (SCD0 condition), there is a varying degree of overlap between STOP and CHANGE task goal processes. Importantly, a temporal overlap of processing several task goals should differentially affect response times given that (dual) task goal processing is subject to capacity limitations and given that task goals which are processed in parallel may furthermore interfere with each other^2^,^3^,^17^,^18^,^19^. Calculating the slope of RTs across SCDs (SCD-RT function) describes how the RT of the response to the CHANGE stimulus varies depending on the delay between STOP and CHANGE stimuli.

The rationale behind this is as follows^2^: The local slope of the SCD function at a given delay (SCD0 and SCD300) reflects the probability that the first process (STOP process) has not finished and overlaps with the following CHANGE process^2^. If the STOP process has not finished, the slope approximates -1. If it has finished, the slope is close to 0^2^. Obtaining a mean slope value in between 0 and -1 hence suggests that the initiation of some (but not all) of the CHANGE responses occurred before the termination of the inhibitory process of stopping the GO response. Hence, the steeper the mean slope, the more likely it is that the STOP process had not finished at the time the CHANGE response was initiated. In other words, the slope of the SCD-RT function is flatter (closer to 0) in case of more serial processing than in case of a more parallel processing mode (where it is closer to -1). In the SCD300 condition, all subjects are forced to serially perform the STOP and the CHANGE process, simply because the temporal gap between the STOP and CHANGE stimuli imposes a cascaded order of task goal processing. However, in the SCD0 condition, the simultaneous presentation of the STOP and CHANGE signals yields the possibility of two different processing modes: a more serial and a more parallel processing mode. Because response selection depends on a restricted resource, the processing mode may differentially affect RT in the SCD0 condition. As long as subjects only vary in the RTs of the SCD0 condition (but not in those of the SCD300 condition, which has repeatedly been demonstrated in previous studies employing the stop change task^3^,^4^,^14^), a distinction between more parallel vs. more serial task goal processing strategies can be made. According to this logic, the ratio (slope) of RTs in the SCD0 condition relative to the SCD300 condition indicates whether a more serial or a more parallel processing mode is in effect. For this calculation, we only used trials in which the initial stopping was successful. No other criteria were used for data inclusion. In order to classify the action cascading strategy applied by each participant, we calculated a general slope value using the mean RTs for both the SCD0 and SCD300 conditions across the entire experiment. We chose to do so across the entire experiment because the slope value used to form the different groups can be seen as a “point estimate” of the subject’s individual strategy used during the task. This estimate is more reliable when all trials of the entire experiment are taken into account. If we had chosen a single bin as the basis to estimate the slope (strategy) this estimate would have necessarily been noisier so that the classification of a subject into a certain group would have been less reliable. Based on the individual slopes, subjects were classified into 2 to 5 different groups. Groups were built with the constraint that group sizes are equal. There were no other criteria. There were also no significant age and sex differences between the different groups (p > .5). The variation in group numbers (2 to 5 groups) was used to examine whether differences were evident when considering opposing extremes of the strategy applied during action cascading. This also avoids the possibility that obtained effects are caused by the artificial group classification. To be able to estimate the response selection mode on a single-trial level, we first calculated the mean RT for responses to CHANGE stimuli in all SCD300 trials for each subject separately. Afterwards, a slope was calculated for each single trial of the SCD0 condition using the RT of this single trial and the mean RT of all SCD300 trials for the respective subject. As a result, we receive a slope value for each SCD0 trial which can be plotted as a function of trials across the experiment. Using this retrospective procedure, it is possible to estimate the strategy used during action cascading on a single trial level.

## Additional Information

**How to cite this article**: Mückschel, M. *et al*. Different strategies, but indifferent strategy adaptation during action cascading. *Sci. Rep.* **5**, 09992; doi: 10.1038/srep09992 (2015).
